# Analysis of the Acute Cytokine Dynamics Induced in Professional Padel According to the Playing Side of the Court and Sex-Related Differences

**DOI:** 10.3390/metabo15060368

**Published:** 2025-06-03

**Authors:** María Pía Cádiz-Gallardo, Francisco Pradas, Pamela Patanè, Alejandro García-Giménez, Miguel Lecina, Luis Carrasco

**Affiliations:** 1 Department of Musical, Plastic and Corporal Expression, Faculty of Health and Sports Sciences, University of Zaragoza, 22001 Huesca, Spain; 838376@unizar.es; 2 Training, Physical Activity and Sports Performance Research Group (ENFYRED), University of Zaragoza, 22001 Huesca, Spain; franprad@unizar.es (F.P.); mlecina@unizar.es (M.L.); 3 Industrial Engineering Department, University of Tor Vergata, 00133 Rome, Italy; pamela.patane@students.uniroma2.eu; 4 Real Club Deportivo Mallorca Performance and Medical Center, 07040 Palma de Mallorca, Spain; 5 Biological and Functional Analysis of Exercise Research Group (BIOFANEX), University of Sevilla, 41004 Sevilla, Spain; lcarrasco@us.es

**Keywords:** racket sports, interleukins, inflammation, immune system, health

## Abstract

**Background/Objectives:** Moderate-intensity physical exercise induces an anti-inflammatory state that may help prevent or manage various diseases. In contrast, high-intensity exercise is closely associated with systemic inflammation, which can lead to immunosuppression, especially when recovery periods are too short, reduced sports performance and potential health risks for the athlete. This study aimed to analyze the acute cytokine dynamics in professional padel players, focusing on differences related to the side of play on the court (forehand or backhand) and sex. **Methods**: A total of 21 elite padel players (11 females and 10 males; age: 27.7 ± 6.3 y) voluntarily participated in the study. Pro-inflammatory cytokines (interleukin (IL)-1ß, IL-2, IL-5, IL-6, IL-7, IL-8, IL-12, tumor necrosis factor alpha and interferon gamma) and anti-inflammatory cytokines (IL-5, IL-6, IL-10, IL-13) were analyzed before and after a padel match. **Results**: The results showed significant changes in pro- and anti-inflammatory, including a decrease in IL-7 (*p* = 0.02), an increase in IL-8 (*p* ≤ 0.001) and an increase in IL-10 (*p* = 0.001). No significant differences were observed based on the side of play on the court, suggesting that this variable does not influence the immune response. **Conclusions**: Competitive padel at an elite level elicits an anti-inflammatory response, characterized by an increase in IL-10 and a reduction in pro-inflammatory cytokines. This response highlights the potential health benefits of padel as a moderate-intensity sport, particularly in managing systemic inflammation.

## 1. Introduction

Studies have demonstrated that exercise of low-to-moderate intensity has been shown to positively influence the immune system by reducing inflammatory responses. These mechanisms are mediated by the enhanced production of anti-inflammatory interleukins such as interleukin-10 (IL-10), interleukin-6 (IL-6), interleukin-13 (IL-13) and interleukin-5 (IL-5) [1]. However, high-intensity exercise or competitive events elicit a pro-inflammatory response, leading to stress-induced immunosuppression that persists over time. This response is associated with tissue damage, including microtraumas and an elevated production of free radicals due to increased oxygen consumption. In this state, the production of interleukin-1 (IL-1), IL-6 (which has both anti- and pro-inflammatory properties), tumor necrosis factor alpha (TNF-α) and interferon-gamma (IFN-γ) is heightened. Consequently, the intensity of the local inflammatory response is proportional to the extent of muscle damage [2,3]. Excessive training loads that induce muscle injury can significantly elevate inflammation, potentially resulting in systemic effects. Such systemic inflammation responses, often part of the acute phase reaction to exercise-related stress, may become intense and prolonged, compromising the athlete’s immune function and exacerbating immunosuppression. These conditions enhance susceptibility to infections, impair performance and represent significant health risks to athletes [4].

To our knowledge, few studies have aimed to analyze the cytokine dynamics during a racket sport competition. In this regard, a study involving young tennis players revealed that during competitive periods, the acute anti-inflammatory immune response was negatively affected, reflected in TNF-α and 1L-ß decreases. However, this response was inverted after three days of rest, during which IL-6 and IL-10 levels augmented [5]. Similarly, Kozlowska et al. [6] investigated the immune response of 38 professional male tennis players across three age categories (cadet, junior and senior) during the competitive season. Their findings indicated increased IL-6 and IL-10 levels (moderate to very large effect sizes) in all groups at mid-season and post-season. TNF-α levels were unclear at mid-season but remained elevated (small effect size) in cadets and seniors post-season.

Another study involving 13 young male badminton players reported significant increases in IL-1α and IL-10 levels at the end of the season compared to pre-season, suggesting an improved ability to respond the inflammatory stimulus given by acute exercise, yet the mechanisms underlying this improvement remain to be unknown [7].

In comparison, padel is a dynamic doubles racket sport that involves short bursts of high-intensity actions, with points lasting 13.5–14.8 s for professional men and 14.4–16.2 s for professional women, comprising 0.80 and 0.73–0.75 shots per second, respectively [8]. These efforts are interspersed with structured recovery intervals, including 20 s rests between points and 90 s breaks between games. This specific game dynamics turns out padel as a moderate intensity demanding sport when referring to physiological indicators as pointed out by Martín-Miguel et al. [8]. In their systematic review, they observed mean heart rate (HR_mean_) values averaging 126.78, 149.0, 159.1 bpm in amateur, semi-professional and professional male players while females averaged ~150 bpm independently of their level. Lactate levels stay at low ranges (1.48 to 3.38 mmol·L^−1^) regardless of gender or level, showing that anaerobic glycolysis plays a minor role. Regarding maximal oxygen consumption (VO_2max_), padel involves effort levels close to 76.3% with spikes reaching 95.1% of VO_2max_ during maximal in-game efforts. Additionally, sex-based differences have been identified in biochemical and hematological biomarkers.

Thus, padel, as a moderate-intensity exercise, could enhance immune function, reinforce antioxidative capacity and reduce oxidative stress, potentially lowering the incidence of inflammation-related diseases [9]. Existing studies on padel have predominantly focused on topics such as performance analysis, psychology, physiology, physical conditioning or medicine among others [10]. However, research on the inflammatory response in padel players remains limited. Pradas et al. [11] analyzed, among other non-immune-related biomarkers, the response of leukemia inhibitory factor (LIF), a multifunctional cytokine related to stem cell renewal and immune modulation, in trained male and female padel players during a competitive match. Their findings revealed no significant increase in LIF release in both male and female players.

Being a doubles racket sport, each player occupies a specific side of the court (right or left) and alternates actions with his/her partner. Previous studies have shown differences in the type and frequency of strokes [12], as well as in the external load metrics [13] depending on the playing side. Besides, not many, but some studies have also shown different physiological within right or left side [14].

Given the increasing popularity of competitive padel and the still remaining unknown of the effect of padel on the immune system, this study aims to analyze the acute inflammatory response during a competitive match, with a specific focus on potential differences based on court side and sex.

## 2. Materials and Methods

### 2.1. Participants

A total of 21 professional padel players (10 males and 11 females) voluntarily participated in this study. The participants were all active competitors on the World Padel Tour, the top-tier international padel circuit, which has since been replaced by the Premier Padel competition. Several of these athletes currently compete on the Premier Padel Tour, maintaining elite-level status. All participants had a minimum of five years of professional experience, ensuring a high level of expertise and familiarity with the sport’s competitive demands. They were young adults between the ages of 18 and 35 years (27.7 ± 6.3 years), a range chosen to focus on athletes in their physical prime. Female players were tested during the early-to-mid follicular phase of their menstrual cycle to minimize the potential influence of hormonal variations on performance and physiological responses.

Participants were excluded if they had injuries or medical conditions that could compromise their performance or interfere with study outcomes. Additionally, those using medications or substances known to affect physiological parameters were not eligible. Detailed characteristics of the participants are presented in Table 1.

The purpose of the study was explained verbally to all participants and informed consent was obtained prior to their inclusion. The research was conducted in accordance with the Declaration of Helsinki [15] and the protocol was approved by the Ethics Committee of the Department of Health than Consumption of the Government of Aragón, Spain (approval code: 21/2012; 19 December 2012).

### 2.2. Experimental Approach

The players were evaluated in two testing sessions, conducted 7 to 9 days apart, between 9:00 a.m. and 12:00 p.m. Prior to each session, participants were instructed to avoid strenuous physical activity for at least 24 h and to adhere to an overnight fasting protocol. Additionally, they were required to abstain from caffeine and alcohol for 12 h before testing. However, players were permitted to hydrate freely during the competition using bottled mineral water.

#### 2.2.1. First Session

Body composition was assessed using bioelectrical impedance analysis (TANITA BC–418MA, Amsterdam, The Netherlands). VO_2max_ and maximum heart rate (HR_max_) were measured through an incremental running test performed on a treadmill (Pulsar HP Cosmos, Nussdorf, Germany) equipped with a gas analyzer (Oxycon Pro, Jaegger, Unterhaching, Germany) and a heart rate monitor (Cosmos, Nussdorf, Germany).

Following a 5 min warm-up at a brisk walking pace (6 km·h^−1^), the treadmill speed was initially set at 8 km·h^−1^ and increased by 1 km·h^−1^ every minute until the participant reached volitional exhaustion. The treadmill slope was maintained at 1% throughout the test. VO_2max_ was determined according to ACSM criteria [16], while HR_max_ was recorded as the highest heart rate achieved during the test.

#### 2.2.2. Second Session

The second session consisted of a simulated padel competition conducted accordance with the rules of the International Padel Federation. Participants were paired based on sex and performance level. Matches were played in a best-of-three sets format; with a tie-break implemented in the event of a 6-6 game score. Prior to each match, players completed a standardized 15 min warm-up. During the competition, players’ heart rates (HR) were continuously recorded at 5 s intervals (Polar Team System, Kempele, Finland). The event took place on outdoor courts under e environmental condition of 24.1 ± 7.1 °C and 45.7 ± 7.3% relative humidity.

### 2.3. Blood Sampling

During the second session, two blood samples of 10 mL each (collected pre- and post-padel competition) were drawn from the antecubital vein of each participant. Each sample contained 35 μmol of EDTA 2K+ and 1500 IU of the kallikrein inactivator. The samples were refrigerated until centrifugation, which was performed at 2150 rpm for 15 min at 4 °C. Plasma aliquots were then separated and stored at −80 °C. Interleukin levels were analyzed using the enzyme-linked immunosorbent assay (ELISA) with the multiplex method, employing the Invitrogen™ Human Cytokine 10-Plex Panel (Thermo Fisher Scientific, Waltham, MA, USA) and read on a Luminex™ platform (Thermo Fisher Scientific, Waltham, MA, USA) according to the manufacturer’s guidelines.

### 2.4. Statistical Analysis

Descriptive and inferential statistical analysis were conducted using the statistical software IBM^®^ SPSS^®^ Statistics (IBM Corp., Armonk, NY, USA) version 30.0 for Windows operating systems. The quantitative variables were described by means of the mean (Mean) and standard deviation (SD) in order to describe their central distribution and variability. The normality of the distributions of the quantitative variables was checked by means of the Shapiro–Wilk test. Parametric analyses were conducted using two-way ANOVA, with side-game and sex as the between-subject factors, to assess intragroup and intergroup differences across laboratory test. The Wilcoxon signed-rank test was used to analyze intragroup differences, while the Mann–Whitney U test was applied to evaluate intergroup differences. Finally, the Kruskal–Wallis test with the respective post hoc contrasts was used to evaluate the differences between groups according to sex and side-game.

In order to consider the observed differences statistically significant, a significance level *p* = 0.05 was adopted. In addition to statistical significance, the effect size (ES) of the observed differences was assessed, using Cohen’s d as an index, with the following interpretations: trivial (d < 0.19), small (d = 0.20), medium (d = 0.50) and large (d = 0.80).

## 3. Results

### 3.1. Participants’ Characteristics and Cardiorespiratory Fitness

As it is shown in Table 1, body composition variables and VO_2max_ exhibited significant sex-related differences (*p <* 0.05). In contrast, age and HR_max_ did not show significant differences.

The two-way ANOVA results from the laboratory tests indicated that female players on the right side (FRS) had a VO_2max_ of 46.2 ± 4.7 mL·kg^−1^·min^−1^, while those on the left side (FLS) exhibited a slightly higher value (48.9 ± 5.1 mL·kg^−1^·min^−1^), with no significant difference between the groups (*p* > 0.05). Conversely, male players on the right side (MRS) exhibited a higher VO_2max_ of 61 ± 6.2 mL·kg^−1^·min^−1^ compared to male players on the left side (MLS), who reached 55.2 ± 4.4 mL·kg^−1^·min^−1^, though the difference was not statistically significant (*p* > 0.05). When all groups were analyzed together, the total VO_2max_ value was 52.2 ± 7.2 mL·kg^−1^·min^−1^, with a significant intergroup difference (*p <* 0.05). Regarding HR_max_, FRS attained 182.7 ± 8 bpm, while FLS had 190.6 ± 5.4 bpm (*p* > 0.05). Similarly, MRS had an HR_max_ of 186.5 ± 8.5 bpm, while MLS showed slightly higher values (189.5 ± 12.5 bpm, *p* > 0.05). The total HR_max_ for all players was 187.2 ± 9.1 bpm, with no significant difference across groups (*p* > 0.05).

### 3.2. Physiological Demands of the Simulated Competition

Female players averaged 149 ± 16 bpm, (80.1% HR_max_ in the lab), which was almost identical to the HR_mean_ achieved by male players (148.9 ± 15.8 bpm; 79.1% HR_max_ in the lab). HR_max_ values were consistent across groups, with female players reaching 171.8 ± 15.3 bpm (92.2% HR_max_ in the lab) and male players 175.9 ± 14.5 bpm (93.46% HR_max_ in the lab). No statistical differences were found between sexes (*p* > 0.05) with ES values close to zero (HR_mean_: −0.006; HR_max_: 0.27), indicating minimal differences between groups.

### 3.3. Analysis of Inflammatory Responses

A moderate and statistically significant negative correlation was observed between body mass index (BMI) and TNF-α levels (r = −0.44; *p* = 0.043). The investigation revealed no statistically significant correlations between the age of the participants and the levels of the cytokines analyzed (Table 2).

Table 2, Table 3 and Table 4 shows biochemical parameters measured including pro-inflammatory (IL-1ß, IL-2, IL-5, Il-6, IL-7, IL-8, IL-12, TNF-α, IFN-γ) and anti-inflammatory cytokines (IL-5, IL-6, IL-10, IL-13) for female, male and total sample, respectively. Notably, IL-6 exhibits both pro-inflammatory and anti-inflammatory effects.

Table 3, Table 4 and Table 5 present the biochemical parameters measured before and after a padel match, including pro-inflammatory (IL-1ß, IL-2, IL-5, IL-6, IL-7, IL-8, IL-12, TNF-α, IFN-γ) and anti-inflammatory cytokines (IL-5, IL-6, IL-10, IL-13). Notably, IL-6 exhibits both pro-inflammatory and anti-inflammatory effects.

No significant changes were observed in female players (Table 3) as all cytokine *p*-values exceeded the significance threshold (*p* > 0.05).

In contrast, male players (Table 4) exhibited significant intragroup differences in three cytokines. IL-7 significantly decreased after the match (*p* = 0.03, ES = 0.67), whereas IL-8 (*p* ≤ 0.00, ES = 0.82) and IL-10 (*p* = 0.01, ES = 0.80) significantly increased.

When analyzing the total sample (Table 5), significant changes were also observed in IL-7 (*p* = 0.02), IL-8 (*p* ≤ 0.00) and IL-10 (*p* = 0.00), reinforcing the trends seen in male players. Additionally, significant sex-based differences were found for IL-8 (*p* = 0.01), suggesting a differential immune response between male and female players.

Finally, Table 6 reflects the results of the unpaired analyses comparing cytokine concentrations between female and male elite padel players before and after the simulated competition. Significant sex differences were observed for IL-8 in the pre-test (*p* = 0.03, ES = 0.99), with higher concentrations in females and for IL-13 in the post-test (*p* = 0.05, ES = 0.65), where males exhibited higher levels. While most other cytokines did not show statistically significant differences, moderate effect sizes were noted for IL-5 (pre-test, ES = 0.71) and IL-13 (pre-test, ES = 0.67).

### 3.4. Analysis According to Playing Side on the Court

An analysis was conducted to examine the influence of playing side on BMI and age. Significant differences in BMI were found when comparing multiple groups based on playing side and sex. Specifically, BMI differed significantly between the FRS and MLS groups (*p* = 0.021) and between the FLS and MRS groups (*p* = 0.011).

**Figure 1:**
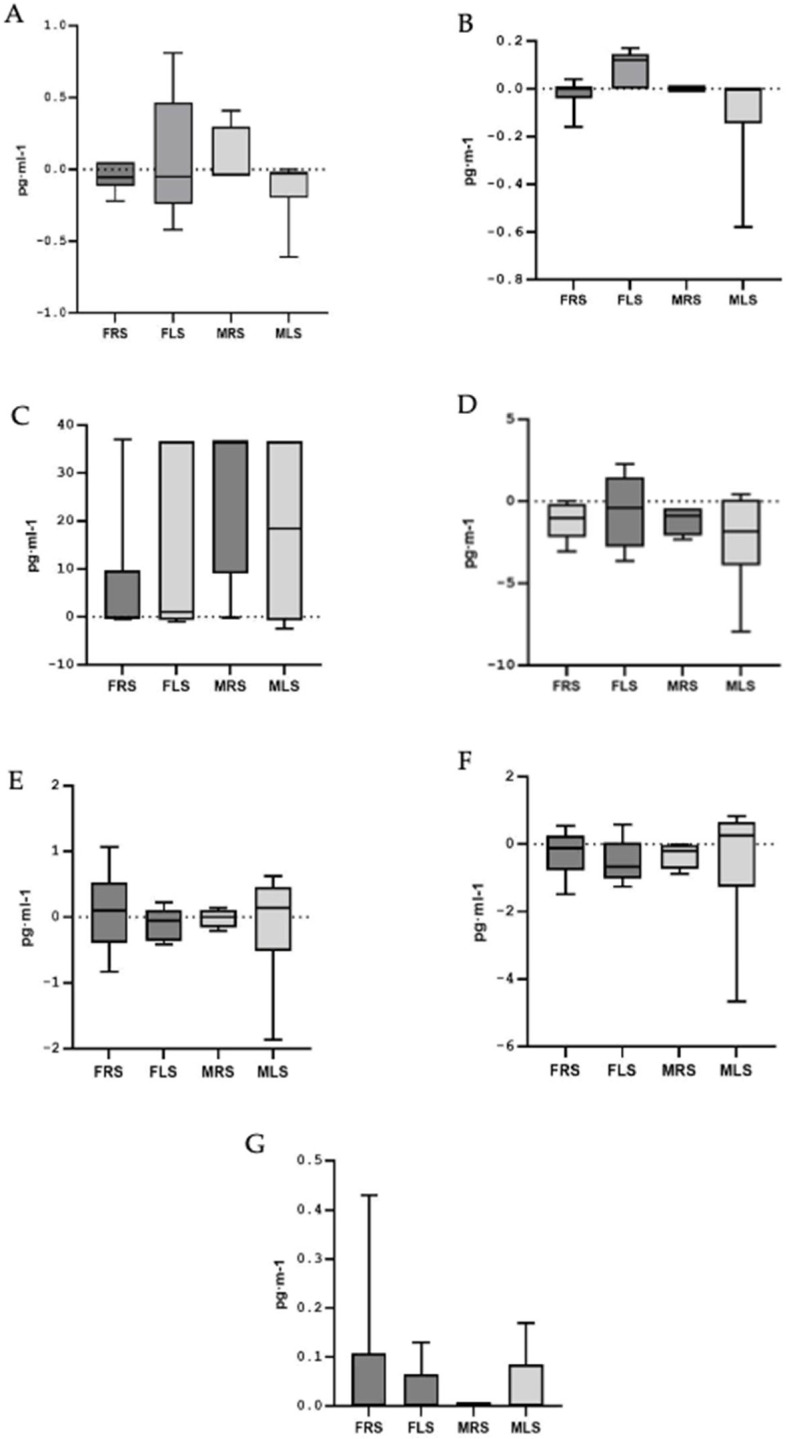
Mean differences in plasma concentration of pro-inflammatory cytokines. (**A**): Interleukin Iβ; (**B**): Interleukin 6; (**C**): Interleukin 7; (**D**): Interleukin 8; (**E**): Interleukin 12; (**F**): Interferon Gamma (IFN-γ); (**G**): Tumor Necrosis Factor Alpha (TNF-α). Note: FRS: female right side; FLS: female left side; MRS: male right side; MLS: male left side.

**Figure 2:**
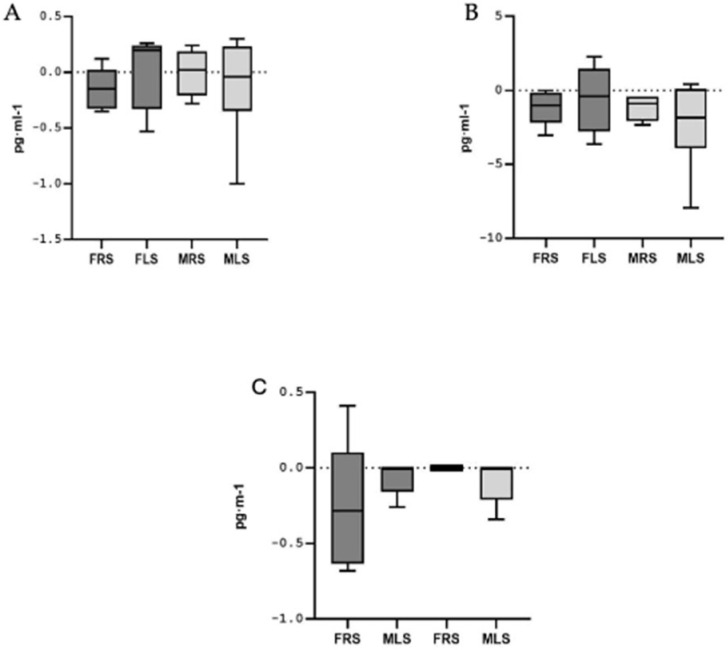
Mean differences in plasma concentration of anti-inflammatory cytokines. (**A**) Interleukin 5; (**B**): Interleukin 10; (**C**): Interleukin 13. Note: FRS: female right side; FLS: female left side; MRS: male right side; MLS: male left side.

To assess potential differences in cytokine responses, a new variable was calculated using the mean differences between pre- and post-test values for each interleukin, categorized by sex and playing side: FRS, FLS, MRS and MLS. The Kruskal–Wallis test (non-parametric) was applied for this analysis. No significant differences in interleukin responses were found between sexes or playing side on the court (see Figure 1 and Figure 2).

## 4. Discussion

Although obesity is typically linked to elevated TNF-α, our negative BMI–TNF-α association likely reflects compensatory anti-inflammatory adaptations in chronically inflamed adipose tissue. Expanding fat depots markedly upregulate IL-10—via M2-like macrophage polarization and STAT3 signaling—which in turn suppresses TNF-α gene expression [17]. Prolonged adipocyte hypertrophy also leads to secretory exhaustion (“adipose tissue failure”), reducing TNF-α output despite high BMI [18]. Clinical reviews further document an increased IL-10/TNF-α ratio in obesity as a homeostatic brake on inflammation [19], and inflamed adipose depots concurrently express other anti-inflammatory mediators (e.g., IL-19), underscoring the shift away from TNF-α secretion in advanced adiposity [20].

Building on these findings, our study also underscores the broader anti-inflammatory shift elicited by padel (↓ IL-7, ↑ IL-10, moderate ↑ IL-8) and situates these changes within a myokine-mediated regulatory network. IL-10, a key anti-inflammatory mediator, signals primarily via STAT3 to upregulate macrophage apoptosis inhibitor and drive M2 macrophage polarization, thereby curbing TNF-α production and promoting tissue repair [21]. Concurrently, IL-8—best known as a neutrophil chemoattractant—acts in a paracrine fashion through CXCR1/2 on endothelial cells to stimulate angiogenesis, facilitating vascular adaptation to muscle work and possibly enhancing nutrient delivery during recovery [22]. IL-7, traditionally viewed in lymphoid homeostasis, has also been shown to promote muscle progenitor activation and hypertrophy in rodents, likely via mTOR-dependent satellite-cell signaling [1].

Beyond these individual roles, exercise-induced reactive oxygen species serve as second messengers that activate PGC-1α isoforms, coordinating mitochondrial biogenesis and antioxidant defenses in muscle fibers [23,24]. In turn, anabolic myokines such as IL-15 reinforce mitochondrial quality control and resilience: IL-15 administration protects myotubes against H_2_O_2_-induced oxidative stress and enhances mitochondrial function via PPARδ-dependent pathways [25]. Finally, several other myokines—including IL-15 and IL-7—have been directly shown to drive muscle hypertrophy in rodent loading models, emphasizing their potential as modulators of exercise adaptation [1].

Together, these data suggest that the cytokine milieu elicited by competitive padel not only tempers pro-inflammatory signals but also engages angiogenic, anabolic and mitochondrial pathways—an integrated response likely crucial for both acute recovery and long-term training adaptations.

This anti-inflammatory profile appears to be closely linked to the specific characteristics of padel as a sport. Padel’s intermittent activity, characterized by short bursts of high-intensity actions (0.73–0.80 shots per second) during short-duration efforts (13.5–16.2 s) during rallies, interspersed with 20 s rest periods and longer 90 s breaks, likely contributes to this response [8]. These activity patterns categorize padel as a low- to moderate-impact sport [8,11,26]. Moderate intensity exercise, as observed in padel, is known to enhance immune function, reinforce antioxidative capacity, reduce oxidative stress and improve energy efficiency, thereby lowering the incidence of inflammatory diseases [9].

Findings from other racket sports align with our results. For instance, Kozłowska et al. [6], who analyzed the effects of tennis match on three groups of high-ranking male players. In the senior group (>18 years), moderate increases in IL-10 and TNF-α were observed, although no significant differences were reported. Similarly, Witek et al. [27] evaluated the influence of tournament workload on IL-6 and irisin in elite young tennis players, considering both singles and doubles matches. Their findings revealed a small effect size for IL-10 increases following seasonal changes. Rossi et al. [7] observed an increase in IL-10 at the end of the annual season with larger increases noted compared to the preseason (+3.4 [0.31 to 5.89] pg·mL^−1^; *p* = 0.047).

This investigation represents the first attempt to analyze the cytokine dynamics during a padel match while examining potential differences based on side of play and sex. No significant differences were found for these factors, suggesting that the side of play is not a major determinant of inflammatory response. Instead, other variables, such as technical, tactical or fitness-related differences, may influence match outcomes. Previous studies have noted that right-side players tend to exhibit higher intensity during rallies, with longer recovery times between points compared to left-side players [28]. On the other hand, Miralles et al. [13] observed a higher frequency of accelerations and decelerations per hour in left-sided players compared to right-sided, potentially leading to distinct physiological demands within the 2 sides.

### 4.1. VO_2max_ and Sex-Related Differences

The VO_2max_ values obtained from the laboratory tests revealed differences between female and male players, with values ranging from 47.4 ± 4.8 and 57.5 ± 5.7 mL·kg^−1^·min^−1^ (*p ≤* 0.01), respectively. These results are consistent with those reported by Pradas et al. [29], who observed values between 46.8 ± 4.6 and 55.4 ± 7 mL·kg^−1^·min^−1^. Similarly, Pradas et al. [11] documented VO_2max_ values of 47.5 ± 4.9 and 57.5 ± 5.7 mL·kg^−1^·min^−1^ for female and male players, respectively. In tennis lower values have been observed for female players (40.9 ± 4.3 mL·kg^−1^·min^−1^) [30], while in badminton, male players demonstrated values of 45.2 ± 8.7 mL·kg^−1^·min^−1^ [31]. The sex-related differences in VO_2max_ may be attributed to physiological factors and match-related variables, such as point duration, match duration and distance covered. García-Benítez et al. [32] reported that female players had longer point durations, more points per game and overall longer match times compared to their male players.

When dividing players by sex and side of play, significant differences in VO_2max_ were noted. FRS had lower values (46.2 ± 4.7 mL·kg^−1^·min^−1^) compared to MRS (61 ± 6.2 mL·kg^−1^·min^−1^; *p* = 0.001) and MLS (55.2 ± 4.4 mL·kg^−1^·min^−1^; *p* = 0.04). FLS also had lower VO_2max_ values (48.9 ± 5.1 mL·kg^−1^·min^−1^) compared to MRS players (61 ± 6.2 mL·kg^−1^·min^−1^; *p* = 0.01). No significant differences were observed between MLS and FLS players. These differences may be influenced by sex, the number of strokes performed per side of play and the distance covered during matches.

### 4.2. Impact of the Playing Side on Heart Rate

Regarding heart rate, our results are consistent with those of Pradas et al. [11] who reported HR_max_ of 186.2 ± 7.8 bpm for males and 183.3 ± 1 bpm in females. HR_mean_ during matches was 72.7 ± 9.8% and 77.2 ± 5.8% HR_max_ for males and females, respectively. In tennis, wider HR_mean_ values have been reported, ranging from 60–80% HR_max_ [33]. In table tennis, slightly lower HR_mean_ values were observed in 60 national level male players (142.69 ± 14.10) [34], while in badminton, higher HR_mean_ values have been reported for both female and male players, ranging from (87.1–93% HR_max_) [35].

In competitive padel matches, no significant differences were found in HR_max_ during laboratory tests or in HR_mean_ during matches based on the side of play. However, right-side players exhibited slightly higher values than left-side players. Similarly, Díaz et al. [36] found higher maximum HR_max_ (181 ± 9.2 vs. 177 ± 10.5 bpm) and HR_mean_ (153 ± 7.8 vs. 137 ± 9.6 bpm) in right-sided semi-professional and amateur female players compared to left sided. These differences could be attributed to variations in the number of actions or strokes performed by players on each side, which may influence cardiovascular demands during matches.

### 4.3. Limitations and New Perspectives

This study is limited by its small sample size (n = 21), which reduces statistical power and generalizability. High interindividual variability in cytokine responses further hindered the identification of consistent patterns. The absence of a control group (e.g., recreational or resting players) limits interpretation of baseline immune responses and the cross-sectional design prevents conclusions about long-term adaptations. Future research should include longitudinal monitoring across the season, integrate hormonal and psychological measures and quantify technical-tactical demands to provide a more comprehensive understanding of cytokine dynamics in elite padel players.

## 5. Conclusions

This study provides empirical evidence that padel practice elicits an anti-inflammatory response, as indicated by a post-exercise decrease in the pro-inflammatory cytokine IL-7 and an increase in the anti-inflammatory cytokine IL-10. A moderate rise in IL-8, which also plays a role in modulating inflammation, further supports this response pattern. These findings suggest that moderate-intensity padel may contribute to immune regulation in trained athletes.

From a theoretical standpoint, these effects could be linked to the moderate oxidative stress induced by padel, promoting a shift toward an anti-inflammatory profile. However, due to the study’s cross-sectional nature and limited sample size, this interpretation should be viewed with caution.

The analysis also explored the role of playing side on immune response, with no significant differences observed between groups. While this supports the notion that playing side may not be a major determinant of inflammatory behavior, further research should investigate more nuanced factors such as workload, hormonal responses and psychological stress.

### Clinical and Practical Implications

These findings may have relevance for coaches, sports medicine professionals and athletes. Given its potential to support an anti-inflammatory profile, padel could be considered a beneficial activity for maintaining immune balance and reducing inflammation-related risks in both athletic and general populations. This may be especially relevant for individuals with higher body mass, as the observed inverse association between BMI and TNF-α suggests possible compensatory immune adaptations. Integrating padel into training or wellness programs might be particularly useful in managing chronic inflammation and promoting overall health, especially when combined with individual monitoring of physiological and psychological markers.

## Figures and Tables

**Table 1:** Padel players’ characteristics.

	Female	Male	Total	*p*	ES
Age (years)	29 ± 3.8	26.3 ± 8.2	27.7 ± 6.3	0.323	−0.42
BMI (kg·m^−2^)	21.7 ± 1	24.4 ± 1.8	23 ± 1.9	0.001	1.85
Weight (kg)	60.6 ± 4.5	76.6 ± 6.2	68.2 ± 9.7	<0.001	2.95
Height (cm)	167.1 ± 5.6	177.1 ± 2.8	171.8 ± 6.7	<0.001	2.25
Body fat (%)	20 ± 2.1	13.3 ± 5.1	16.9 ± 5.1	0.001	−1.71
Muscle mass (%)	37 ± 2.9	43.3 ± 2.2	27.5 ± 5.8	<0.001	2.44
VO_2max_ (mL·kg^−1^·min^−1^)	47.4 ± 4.8	57.5 ± 5.7	52.81 ± 7.2	<0.001	1.91
HR_max_ (bpm)	186.2 ± 7.7	188.2 ± 10.6	187.2 ± 0.9	0.639	0.22

Notes: values are presented as mean ± standard deviation; *p* = *p*-value; ES = effect size (Cohen’s d); BMI = body mass index; VO_2max_ = maximum oxygen consumption; HR_max_ = maximum heart rate measured in the graded exercise test.

**Table 2:** Correlation between mean difference of cytokines, BMI and age.

		IL-1ß	IL-2	IL-5	IL-6	IL-7	IL-8	IL-10	IL-12	IL-13	TNF-α	IFN-γ
BMI	*r*	−0.33	0.40	−0.33	−0.39	0.75	0.10	−0.40	−0.21	−0.27	−0.44 *	−0.33
	*p*	0.13	0.86	0.88	0.07	0.74	0.65	0.07	0.34	0.22	0.043 *	0.13
Age	*r*	0.11	0.08 ^+^	−0.27	0.26	0.57	0.02 ^+^	0.21	0.29	0.01	0.71	0.22
	*p*	0.61	0.70	0.23	0.25	0.80	0.92	0.34	0.20	0.96	0.75	0.33

BMI = body max index; *r* = Pearson’s correlation; * *p* < 0.05 indicates statistical significance; ^+^ = U statistic from Mann–Whitney U test (non-parametric).

**Table 3:** Female’s pro-inflammatory and anti-inflammatory cytokines values measured before (pre) and after (post) a competition padel match.

Cytokines (pg·mL^−1^)	Pre		Post		Intragroup Contrast		
	Mean ± SD	Range	Mean ± SD	Range	*p*	ES	Z
IL1-ß	0.41 ± 0.23	0.06–1.01	0.40 ± 0.09	0.20–0.49	0.32	0.34	−1.721 ^a^
IL-2	0.70 ± 0.40	0.09–1.52	0.76 ± 0.29	0.09–1.11	0.38	0.26	0.866 ^a^
IL-5	0.81 ± 0.53	0.00–1.99	0.88 ± 0.49	0.17–1.87	0.32	0.24	−0.798 ^a^
IL-6	0.39 ± 0.70	0.18–2.50	0.16 ± 0.82	0.01–0.34	0.22	0.37	−1.214 ^a^
IL-7	10.67 ± 16.91	0.16–37	0.72 ± 0.41	0.00–1.46	0.28	0.32	−1.071 ^b^
IL-8	1.20 ± 0.80	0.34–3.28	1.40 ± 1.03	0.00–3.93	0.09	0.50	−1.682 ^a^
IL-10	4.11 ± 2.48	0.31–6.67	5.04 ± 2.68	0.99–10.30	0.11	0.47	−1.580 ^a^
IL-12	0.91 ± 0.52	0.36–1.68	0.92 ± 0.63	0.23–2.19	0.76	0.89	−2.96 ^a^
IL-13	0.57 ± 0.42	0.24–1.43	0.73 ± 0.57	0.24–1.60	0.09	0.51	−1.690 ^a^
IFN-γ	3.17 ± 1.84	0.47–7.14	3.54 ± 1.76	0.61–6.56	0.13	0.47	−1.511 ^a^
TNF-α	0.43 ± 0.00	0.43–0.43	0.37 ± 0.13	0.00–0.43	0.18	0.40	−1.342 ^b^

SD = standard deviation; *p* = *p*-value; ES = effect size (Cohen’s d); Z = z-score; ^a^ based on negative ranks; ^b^ based on positive ranks.

**Table 4:** Male’s pro-inflammatory and anti-inflammatory cytokines values measured before (pre) and after (post) a competition padel match.

Cytokines (pg·mL^−1^)	Pre		Post		Intragroup Contrast		
	Mean ± SD	Range	Mean ± SD	Range	*p*	ES	Z
IL1-ß	0.24 ± 0.21	0.00–0.62	0.28 ± 0.35	0.06–1.23	0.08	0.54	−1.721 ^a^
IL-2	0.55 ± 0.62	0.03–2.11	0.57 ± 0.78	0.09–2.74	0.95	0.16	0.59 ^a^
IL-5	0.54 ± 0.34	0.17–1.31	0.61 ± 0.63	0.09–1.87	0.88	0.04	−0.14 ^a^
IL-6	0.20 ± 0.06	0.18–0.38	0.25 ± 0.24	0.18–0.96	0.31	0.31	−1.000 ^a^
IL-7	22.51 ± 18.70	0.16–37	0.78 ± 1.35	0.29–4.64	0.03 *	0.67	−2.140 ^a^
IL-8	0.61 ± 0.24	0.22–0.98	0.81 ± 0.40	0.30–1.75	≤0.00 *	0.82	−2.599 ^a^
IL-10	2.37 ± 2.44	0.54–8.70	4.20 ± 4.45	1.44–16.62	0.01 *	0.80	−2.547 ^a^
IL-12	0.68 ± 0.69	0.15–2.39	0.75 ± 1.25	0.07–4.26	0.61	1.60	−5.07 ^b^
IL-13	0.44 ± 0.53	0.24–1.89	0.62 ± 0.98	0.24–3.34	0.18	0.42	−1.342 ^a^
IFN-γ	2.23 ± 2.25	0.13–6.27	2.64 ± 3.21	0.13–10.48	0.08	0.21	0.67 ^a^
TNF-α	0.43 ± 0.00	0.43–0.43	0.44 ± 0.05	0.43–0.60	0.317	0.82	−2.599 ^a^

SD = standard deviation; *p* = *p*-value; ES = effect size (Cohen’s d); Z = z-score; * *p* < 0.05 indicates statistical significance; ^a^ based on negative ranks; ^b^ based on positive ranks.

**Table 5:** Total players’ pro-inflammatory and anti-inflammatory cytokines values measured before (pre) and after (post) a competition padel match.

Cytokines (pg·mL^−1^)	Pre		Post		Intragroup Contrast			Sex Intergroup			
	Mean ± SD	Range	Mean ± SD	Range	*p*	ES	Z	*p*	ES	*p*	ES
IL1-ß	0.33 ± 0.23	0.0–1.01	0.34 ± 0.25	0.06–1.23	0.15	0.25	−1.149 ^a^	0.13	0.71	0.02	1.12
IL-2	0.63 ± 0.51	0.03–2.11	0.67 ± 0.57	0.09–2.74	0.49	0.14	−0.684 ^a^	0.25	0.54	0.01	1.20
IL-5	0.68 ± 0.46	0.0–1.99	0.75 ± 0.56	0.09–1.87	0.38	0.19	−0.865 ^a^	0.19	0.61	0.13	0.71
IL-6	0.30 ± 0.50	0.18–2.5	0.20 ± 0.18	0.01–0.96	0.60	0.17	−0.524 ^b^	1.00	0	0.19	0.61
IL-7	16.31 ± 18.36	0.16–37	0.75 ± 0.95	0.0–4.64	0.02 *	0.48	−2.219 ^b^	0.31	0.47	0.05	0.95
IL-8	0.92 ± 0.66	0.22–3.28	1.12 ± 0.83	0.0–3.93	≤0.00 *	0.64	−2.949 ^a^	0.01	1.22	0.08	0.83
IL-10	3.28 ± 2.55	0.31–8.7	4.64 ± 3.56	0.99–16.62	0.00 *	0.61	−2.817 ^a^	0.11	0.73	0.11	0.75
IL-12	0.80 ± 0.60	0.15–2.39	0.83 ± 0.95	0.07–4.26	0.91	0.02	−0.103 ^b^	0.28	0.49	0.04	0.97
IL-13	0.30 ± 0.46	0.24–1.89	0.68 ± 0.77	0.24–3.34	0.38	0.45	−2.073 ^a^	0.15	0.66	0.15	0.68
IFN-γ	2.72 ± 2.05	0.13–7.14	3.11 ± 2.53	0.13–10.48	0.15	0.30	−1.419 ^a^	0.19	0.61	0.19	0.74
TNF-α	0.43 ± 0.00	0.43–0.43	0.41 ± 0.10	0.0–0.6	0.59	0.12	−0.535 ^a^	1.00	0	0.31	0.46

SD = standard deviation; *p* = *p*-value; ES = effect size (Cohen’s d); Z = z-score; * *p* < 0.05 indicates statistical significance; ^a^ based on negative ranks; ^b^ based on positive ranks.

**Table 6:** Comparison of cytokine concentrations (pg·mL^−1^) between female and male elite padel players before (Pre-test) and after (Post-test) the intervention. Values are presented as Mean ± Standard Deviation (SD). Between-group differences were assessed using independent samples *t*-tests (parametric data) or Mann–Whitney U tests (non-parametric data), as appropriate.

Pre Test							Post Test					
Cytokines (pg·mL^−1^)	Female Mean ± SD	Male Mean ± SD	t/U ^†^	df/Z ^‡^	*p*	ES	Female Mean ± SD	Male Mean ± SD	t/U ^†^	df/Z ^‡^	*p*	ES
IL1-ß	0.41 ± 0.23	0.24 ± 0.21	0.0	19	1	0.77	0.40 ± 0.91	0.28 ± 0.35	1.10	19	0.28	−0.17
IL-2	0.70 ± 0.40	0.55 ± 0.62	38 ^†^	−1.19 ^‡^	0.23 ^†^	0.28	0.76 ± 0.29	0.57 ± 0.78	0.78	19	0.44	−0.32
IL-5	0.81 ± 0.53	0.54 ± 0.34	36 ^†^	−1.34 ^‡^	0.17 ^†^	0.60	0.88 ± 0.49	0.61 ± 0.63	33 ^†^	−1.55 ^‡^	0.12 ^†^	−0.47
IL-6	0.19 ± 0.036	0.20 ± 0.64	−0.41	19	0.68	0.02	0.16 ± 0.82	0.25 ± 0.24	−1.18	19	0.25	0.14
IL-7	10.45 ± 17.04	22.37 ± 18.88	−1.52	19	0.14	0.66	0.72 ± 0.41	0.39 ± 0.12	2.47	12.06	0.02 *	−1.02
IL-8	1.20 ± 0.80	0.61 ± 0.24	2.22	19	0.03 *	0.99	1.40 ± 1.03	0.81 ± 0.40	30 ^†^	−1.76 ^‡^	0.78 ^†^	−0.75
IL-10	4.11 ± 2.48	2.37 ± 2.44	1.16	19	0.12	0.70	5.04 ± 2.68	4.20 ± 4.45	0.52	19	0.60	−0.22
IL-12	0.91 ± 0.52	0.68 ± 0.69	0.87	19	0.39	0.37	0.92 ± 0.63	0.75 ± 1.25	0.39	19	0.69	−0.17
IL-13	0.57 ± 0.42	0.30 ± 0.14	1.95	19	0.07	0.86	0.73 ± 0.57	0.35 ± 0.26	30 ^†^	−1.95 ^‡^	0.05 *^†^	0.85
IFN-γ	3.54 ± 1.76	2.64 ± 3.21	36 ^†^	−133 ^‡^	0.19 ^†^	0.34	3.54 ± 1.76	2.64 ± 3.32	32	−1.58	0.11	0.33
TNF-α	0.43 ± 0.0	0.43 ± 0.0	0.0 ^†^	18.9 ^‡^	1 ^†^	0.0	0.37 ± 0.13	0.44 ± 0.54	−1.51	19	0.14	0.96

t = *t*-test; U = Mann–Whitney U; df = degrees of freedom from *t*-test; Z = z-score; *p* = *p* value; ES = effect size (Cohen’s *d*); ^†^ = U statistic from Mann–Whitney U test was used (non-parametric); ^‡^ = z-score was used (non-parametric); * *p* < 0.05 indicates statistical significance.

## Data Availability

The original contributions presented in this study are included in the article. Further inquiries can be directed to the corresponding author.

## Abbreviations

BMI	Body mass index
ES	Effect size (ES)
FLS	Female players on the left side (FLS)
FRS	Female players on the right side (FRS)
HR_max_	Maximum heart rate
HR_mean_	Mean heart rate
IFN-γ	Interferon-gamma
IL-1	Interleukin-1
IL-10	Interleukin-10
IL-13	Interleukin-13
IL-5	Interleukin-5
IL-6	Interleukin-6
LIF	Leukemia inhibitory factor
MLS	Male players on the left side (MLS)
MRS	Male players on the right side (MRS)
SD	Standard deviation
TNF-α	Tumor necrosis factor alpha
VO_2max_	maximal oxygen consumption
